# Non-Pharmacological Approaches to Headaches: Non-Invasive Neuromodulation, Nutraceuticals, and Behavioral Approaches

**DOI:** 10.3390/ijerph18041503

**Published:** 2021-02-05

**Authors:** Licia Grazzi, Claudia Toppo, Domenico D’Amico, Matilde Leonardi, Paolo Martelletti, Alberto Raggi, Erika Guastafierro

**Affiliations:** 1 UOC Neuroalgologia, Fondazione IRCCS Istituto Neurologico Carlo Besta, 20133 Milan, Italy; domenico.damico@istituto-besta.it; 2 UOC Neurologia, Salute Pubblica e Disabilità, Fondazione IRCCS Istituto Neurologico Carlo Besta, 20133 Milan, Italy; claudia.toppo@istituto-besta.it (C.T.); matilde.leonardi@istituto-besta.it (M.L.); alberto.raggi@istituto-besta.it (A.R.); erika.guastafierro@istituto-besta.it (E.G.); 3 Department of Clinical and Molecular Medicine, Sapienza University, 00185 Rome, Italy; paolo.martelletti@uniroma1.it

**Keywords:** non-invasive neurostimulation, nutraceuticals, behavioral approaches, headache

## Abstract

Significant side effects or drug interactions can make pharmacological management of headache disorders very difficult. Non-conventional and non-pharmacological treatments are becoming increasingly used to overcome these issues. In particular, non-invasive neuromodulation, nutraceuticals, and behavioral approaches are well tolerated and indicated for specific patient categories such as adolescents and pregnant women. This paper aims to present the main approaches reported in the literature in the management of headache disorders. We therefore reviewed the available literature published between 2010 and 2020 and performed a narrative presentation for each of the three categories (non-invasive neuromodulation, nutraceuticals, and behavioral therapies). Regarding non-invasive neuromodulation, we selected transcranial magnetic stimulation, supraorbital nerve stimulation, transcranial direct current stimulation, non-invasive vagal nerve stimulation, and caloric vestibular stimulation. For nutraceuticals, we selected Feverfew, Butterbur, Riboflavin, Magnesium, and Coenzyme Q10. Finally, for behavioral approaches, we selected biofeedback, cognitive behavioral therapy, relaxation techniques, mindfulness-based therapy, and acceptance and commitment therapy. These approaches are increasingly seen as a valid treatment option in headache management, especially for patients with medication overuse or contraindications to drug treatment. However, further investigations are needed to consider the effectiveness of these approaches also with respect to the long-term effects.

## 1. Introduction

Headache disorders are common disabling conditions and include tension-type headache (TTH) and migraine [1]. Most persons have experienced at least one episode of headache in their life course. Indeed, headache disorders are the second and third most prevalent disorders in the world [1]: 62.2% of the adult population is affected by episodic tension-type headache (TTH), 14.7% by episodic migraine, 3.3% by chronic tension-type headache (CTTH), and 2% by chronic migraine (CM). In a few studies carried out on the pediatric population, it is possible to observe a lower prevalence of these diseases, which, however, remains quite high (9.2% for migraine, 15.9% for TTH, and 0.9% for CTTH) [2,3,4].

Headaches, and migraines in particular, are complex diseases in which psychological, social, and biological aspects are closely connected. In this perspective, a biopsychological intervention seems the most appropriate to manage this condition adequately and this is determined by different components: proper diagnosis, patient education, multidimensional assessment, strategic intervention including different approaches, according to the assessment and patient’s needs, changes monitoring, and follow-up [5,6].

Headache management includes drug treatments such as non-steroidal anti-inflammatory drugs, triptans, as well as prophylactic drugs (e.g., amitriptyline, topiramate, onabotulinum toxin-A, and β-blockers), and new drugs such as anti-CGRP monoclonal antibodies [7]. The phenomena of tolerance and pharmacoresistance to common preventive pharmacological therapies can make the pharmacological treatment of chronic headaches complex [8], which is sometimes associated with side effects and complex interaction between different drugs.

Therefore, new headache treatment options need to be found. In recent decades, innovative non-invasive neuromodulatory techniques, for example, transcranial magnetic stimulation (TMS) and supraorbital nerve stimulation (SNS), which have shown remarkable results, have been developed [9]. In recent years, complementary and alternative medicine (CAM) has been found to be a compelling option for headache disorders [10,11]. CAM includes several practices and products of the health care system, which are considered complementary or supplementary to conventional techniques and therapies. Finally, behavioral approaches are emerging as promising treatments to change maladaptive behavior and ways of thinking that could enhance headache-related burden and pain.

Within a biopsychosocial approach, a multidisciplinary program for headache management should be planned. It should include not only conventional pharmacological therapies but also: patient education and support, lifestyle modification (diet, physical activity, lifestyle habits, stressful conditions, etc.), and complementary measures. These may include nutraceuticals, neuromodulation techniques, behavioral approaches, and psychotherapy intervention when necessary [12,13,14].

Non-pharmacological interventions may be useful to help patients manage pain and they are usually well tolerated. The use of these methodologies is suggested for specific categories of patients, such as pregnant women and adolescents, and also when the use of drugs must be limited or avoided due to contraindications, excessive use, or known side effects. [4,15,16,17,18,19].

The aim of the present issue is to discuss non-pharmacological approaches, including recent studies that demonstrate applications of these techniques on patients with different diagnoses of headache. Particularly, we will focus on: non-invasive neuromodulation, such as non-invasive vagal nerve stimulation and transcranial direct cortical stimulation; nutraceuticals, which comprise various types of products, including isolated nutrients and food supplements (e.g., minerals/vitamins and herbal remedies) which are not considered conventional medical practices; behavioral approaches, such as biofeedback and cognitive behavioral therapy.

## 2. Materials and Methods

We reviewed the available literature on non-pharmacological approaches to headache, reporting articles focusing on: non-invasive neuromodulation, i.e., transcranial magnetic stimulation (TMS), supraorbital nerve stimulation (SNS), transcranial direct current stimulation (tDCS), non-invasive vagal nerve stimulation (nVNS), and caloric vestibular stimulation (CVS); nutraceuticals, i.e., Butterbur, Riboflavin, Magnesium, and Coenzyme Q10; behavioral approaches, i.e., biofeedback, relaxation techniques, cognitive behavioral therapy (CBT), mindfulness-based therapy (MBT), and acceptance and commitment therapy (ACT).

We focused our search to reviews and original studies published from 2010 to 2020 in order to refer to recent data. For each category, a narrative presentation will be made of the approaches published in the period of interest, with particular focus on the primary outcomes reported in the various studies.

## 3. Results

### 3.1. Non-Invasive Neuromodulation

Although neuromodulation techniques were initially invasive, advances and progress in technology have led to more sophisticated and less invasive devices with better safety and efficiency [9].

The first neuromodulation approach analyzed in this review used deep brain stimulation (DBS), targeting the hypothalamus, for cluster headaches [20]. Subsequently, to reduce the risk and invasiveness of neuromodulation, the sphenopalatine ganglion [21,22,23] and the occipital [24,25], supraorbital, and auriculo-temporal [22] nerves were targeted, in cluster headaches and also in migraines. With technological innovations, which have led to further improvements of these techniques in terms of efficacy, safety, and reduced invasiveness, it has been possible to use neuromodulation even in patients who had comorbidities or contraindications to traditional pharmacological treatment.

In the following sections, a description of some non-invasive neuromodulation devices will be discussed.

#### 3.1.1. Transcranial Magnetic Stimulation (TMS)

TMS is a neuromodulatory technique whose mechanism of operation is not fully understood yet. It seems to consist of the induction of electric current in the brain through an external pulsed magnetic stimulation. It is thought that TMS induces electric fields in the brain that cause a flow of electric currents which, when they have reached adequate size, position, and duration, depolarize the neural tissue, generating an action capable of influencing cortical excitability. Cerebral cortex excitability modulation, induced by TMS, has been used both for the management of chronic migraines and migraines with and without aura [26,27].

There are two main working manner of the TMS: single-pulse stimulation or repetitive pulse stimulation.

##### Single Pulse Transcranial Magnetic Stimulation (sTMS)

Clarke [28] and subsequently, Lipton [29] reported relevant clinical experiences of single pulse TMS (sTMS) for migraine treatment.

The first study [30] reported the efficacy of two pulses of sTMS at either low or high intensity, in the treatment of an attack of migraine without aura, applying stimulation over the painful region of the scalp, or of an attack of migraine with aura, positioning the stimulus over the visual cortex region in the treatment. This study produced encouraging results, with minimal adverse events: participants described a reduction in pain intensity of 75% for up to 20 min post stimulation. In addition, about 1/3 of patients showed no headache attack for up to 24 h after one treatment.

Lipton and colleagues [29] conducted a study positioning an sTMS device over the visual cortex in migraine with aura. Efficacy over the placebo was demonstrated: patients reported pain freedom 2 h after treatment (39% vs. 22%).

Other studies [30,31], conducted on episodic migraine and CM, presented encouraging results such as reduction in headache days per month after a period of neurostimulation treatment. This may suggest the use of sTMS in headache prevention treatments.

##### Repetitive Transcranial Magnetic Stimulation (rTMS)

rTMS has been tested in several studies for the prevention of both episodic migraine and CM. In 2013, Misra and colleagues demonstrated [32] with a randomized placebo-controlled study the prophylactic benefit of rTMS for CM patients, which reported minimal adverse events.

Other randomized control groups and double-blind studies showed encouraging results with minimal side effects [33,34]. One study, however, highlighted minimal adverse events with rTMS such as transient drowsiness, reported in one patient [32]. Unfortunately, the variability in stimulation parameters, location, and protocols among the studies do not allow for generalizability of the results. Furthermore, the reliability of these studies has been reduced due to the short follow-up periods although, in general, the results generated by these studies seem encouraging. [35].

#### 3.1.2. Supraorbital Nerve Stimulation (SNS)

The supraorbital and supratrochlear nerves are terminal branches of the frontal nerve, derived from the ophthalmic division of the trigeminal nerve; they provide sensation to the area of the forehead and the upper eyelid. Stimulation of these nerves can be used to treat migraine. This treatment is probably effective due to the inhibition of nociceptive transmission in the small pain transmitting fibers, producing the modulation of nociceptive activity in the trigeminal ganglion [36].

Originally, SNS was an invasive technique used for the treatment of cluster headache with encouraging results [37,38]. Other studies demonstrated notable results of SNS in the treatment of both episodic migraine and CM [25,39].

The Cefaly^®^ device is a new non-invasive transcutaneous supraorbital device, developed in recent years, which appears to have several useful applications for the treatment of headache [40]. This device mechanism consists of a transcutaneous transmission, through an adhesive supraorbital electrode, which excites the supratrochlear and supraorbital branches of the ophthalmic nerve in the forehead [41]. This device is used directly at home by trained patients who use it for a 20-min daily session or they can also use it during an acute migraine attack.

Several studies demonstrated its efficacy in migraine management, resulting in a reduction in the mean number of migraine attack per month, with a significant reduction in acute medication intake and minimal side effects [42,43]. Cefaly^®^ was the first device to be FDA approved, in March 2014, for the preventive management of episodic migraine [40], resulting as a suitable alternative to medications. Despite these studies showing its effectiveness, patients that use the device reported issues of adherence. For example, in the study of Schoenen [40], discontinuation from treatment was due to onset of paresthesia and, if no benefit was recorded in a few weeks, patients stopped daily application of the device earlier than the scheduled time of 3 months. Therefore, it is necessary to manage the device, providing adequate patient education and reinforcement [40].

#### 3.1.3. Transcranial Direct Current Stimulation (tDCS)

tDCS is a central, non-invasive neurostimulation technique which has the advantage of being portable and less expensive compared to other devices used for non-invasive neurostimulation [40]. tDCS modifies the resting membrane potential of cortical neurons through a weak current, inducing a modulation of cortical excitability [40]. To date, tDCS has been used in two different modalities: cathodal and anodal. The first one seems to decrease cortical excitability while the second one seems to increase cortical excitability [44].

Two studies on migraine patients, carried out in 2011 [45] and 2015 [46], respectively, applied cathodal stimulation to reduce cortical excitability. Both these studies failed in showing the efficacy of this technique.

Regarding anodal stimulation, used to increase cortical excitability, two small controlled studies by Auvichayapat [47] and by Da Silva [48] tried to evaluate its efficacy in episodic and chronic migraine, respectively. These two studies have reported both a reduction in the intensity and duration of attacks and a decrease in medication intake. An 8-week pilot study, which demonstrated the efficacy of anodal tDCS, especially in treatment of migraine without aura, was conducted in 2013 [49].

A study that considered both anodal and cathodal modality, conducted in 2020 [50], demonstrated that tDCS is not effective in patients with CM and medication overuse after withdrawal, reporting the same results between the group treated with tDCS and the control group at one-year follow up.

Despite the encouraging results reported by these studies, it must be taken into account that they are preliminary and should be evaluated with caution. However, in general, the adverse effects of tDCS are mainly quite mild and reversible: patients might experience tingling, itching, pain in the neck or scalp, redness of the skin, rash, or minor burns after application [44].

#### 3.1.4. Non-Invasive Vagal Nerve Stimulation (nVNS)

VNS is a surgical procedure used in studies on comorbid migraine in refractory epilepsy [51,52] but it also seems useful in migraine management [53,54]. In subsequent studies, the efficacy of VNS has been shown in the treatment of intractable migraine and cluster headache in patients with depression [55,56].

Recently, a novel non-invasive handheld vagal nerve stimulation (nVNS) technique has been developed. The utility and benefits of nVNS are demonstrated by several studies, especially for the acute treatment of migraine and of cluster headache [57,58]. In one of these studies, patients with episodic migraine with and without aura and patients with CM or high frequency migraine without aura showed a 2-h pain-free state after treatment [59]. Greater efficacy of nVNS was observed in a study with high-frequency migraine patients compared to CM patients: in the first group, 78.6% of patients reported pain relief while in the second one, only 55.9% of patients reported pain relief.

The PRESTO study proposed nVNS as an acute treatment for migraine, showing greater efficacy of nVNS, compared to the sham condition, in reducing a migraine attack [60]. The efficacy of the Gammacore^®^ nVNS device was also demonstrated by an open-label study [61]. The device was used as a mini-prophylaxis in a problematic category of patients, with menstrual migraine or menstrual-related migraine, that generally failed with pharmacological treatments The results showed that using the device in bilateral stimulations for two minutes three times a day, patients reported a significant reduction in migraine days from 7.2 to 4.7 days per month, using the device from 3 days before menstruation to up to 3 days after.

Another pilot study, called EVENT [62,63], showed encouraging results: in the prophylaxis of CM, the use of nVNS produced a similar decrease in both study groups at the end of the double-blind period. Although this study confirmed that greater reductions in the number of headache days are associated with longer treatment periods, it is not clear how long the treatment period would have to last to achieve a neuromodulation effect. Therefore, the effectiveness of nVNS in chronic migraine still remains an unanswered question.

#### 3.1.5. Caloric Vestibular Stimulation (CVS)

CVS has recently been tested as a support therapy for the prevention of episodic migraine. CVS comprises a change in temperature, due to irrigation with water of the external auditory canal, which evokes a slow-phase nystagmus towards the ear causing sensations of virtual rotation of the body and dizziness. A multicenter clinical trial [64] conducted on patients with episodic migraine followed for a period of 3 months produced good results in terms of fewer migraine days and medication intake compared to placebo. The mechanism of action of CVS produces encouraging results as it aims at neuromodulation of the brain stem due to changes in cerebrovascular dynamics and pain relief given by caloric irrigation [65,66].

#### 3.1.6. Non-Painful Remote Electrical Skin Stimulation

Non-painful remote electrical skin stimulation consists of skin electrodes applied to the upper arm to stimulate peripheral nerves. The mechanism of action consists of inducing conditioned pain modulation that inhibits pain in remote body regions [65,67]. This technique was implied in a study at migraine onset for 20 min. Results showed a 50% of pain reduction in the majority of patients (64%) compared to the sham condition. The conditioned pain modulation effect activates the descending pathways of inhibition, thus putting the working mechanism in place [68].

### 3.2. Nutraceuticals

Nutraceuticals include “natural” substances such as vitamins, minerals, and herbal remedies that may be less toxic than drugs. Some of these remedies appear to be effective and promising in the management of chronic pain but most current studies are underpowered or show inconsistent results [19]. Recently, the use of nutraceuticals has been expanding as an aid in the treatment of headache disorders in adults who prefer these approaches over traditional drug remedies.

Guidelines from the American Academy of Neurology (AAN), American Headache Society (AHS), the Canadian Headache Society (CHS), and the European Federation of Neurological Societies (EFNS) discussed the use of nutraceuticals in migraine prophylaxis, sometimes reporting conflicting recommendations [18].

Among the most common nutraceuticals used for headache and migraine, we found different compounds and a brief description will be in the following section.

#### 3.2.1. Feverfew (*Tanacetum Parthenium*)

Feverfew is an herbal preparation used in the treatment of fevers, toothache, headache, arthritis, and inflammation. Its antimigraine properties are probably due to the parthenolide present in the leaves of the *Tanacetum parthenium* plant. Parthenolide prevents migraine through an antiplatelet mechanism that leads to vascular smooth muscle relaxation and an anti-inflammatory mechanism which implies the inhibition of prostaglandin synthesis and phospholipase A [68,69,70,71,72]. These mechanisms seem to influence the pathogenesis of migraine and this is why Feverfew is one of the most tested nutraceuticals in the field of migraine [17].

The results on the use of Feverfew are still controversial: several randomized controlled trials have shown encouraging results in migraine treatment [73,74]; on the other hand, however, there have been many studies that have not registered its efficacy [75,76,77]. This is probably due to heterogeneity in dosage, patient groups, methodologies, and collection of information. Therefore, the differences discovered in the literature makes a definite conclusion on the use of feverfew in migraine treatment challenging.

#### 3.2.2. Butterbur (*Petasites Hybridus*)

Butterbur is a perennial shrub whose mechanism includes an anti-inflammatory action that implies the inhibition of cyclooxygenase-2 [78] and vasodilatory effects given by the inhibition of L-type voltage-gated calcium channels [79]: this anti-inflammatory action allows the use of Butterbur in migraine prophylaxis. 

In a 16-week trial, conducted by Lipton and colleagues [80], three groups of treatment were compared: Butterbur 50 mg twice daily, Butterbur 75 mg twice daily, and placebo. The 75 mg Butterbur group reported a significant reduction in migraine attacks per months (−45%), while the other two groups reported a minor decrease in the number of migraine attacks per month (−32% for the 50mg Butterbur group and –28% for the placebo group).

Even other research studies, conducted to understand the efficacy of Butterbur in preventing migraine in adults and children, showed encouraging results [81,82,83,84]. However, there are also studies reporting possible toxicity and safety of Butterbur, particularly regarding hepatic toxicity [85,86], prompting scientists to recommend attention in its use.

#### 3.2.3. Riboflavin (Vitamin B2)

Riboflavin is a member of the vitamin B2 family, which plays a key role in the production of cellular energy: its mechanism acts as a cofactor for flavoprotein enzyme function in the electron transport chain of the Krebs cycle, stabilizing and maintaining energy-related cellular functions [68]. Riboflavin is thus important in the mitochondrial energy metabolism effective for migraine.

Schonen and colleagues [87] conducted a study that compared treatment with 400 mg riboflavin daily with placebo for 12 weeks. The results, 4 months after the trial, showed that the reduction in migraine attacks per month was greater for patients treated with riboflavin (56%) compared to the placebo group (19%).

Several studies reported encouraging results on the use of Riboflavin as it seems to be well tolerated by adults with migraine and to be safe and effective, encouraging its use in migraine prevention [87,88].

#### 3.2.4. Magnesium

Magnesium appears to be essential in regulating various vascular and neuronal mechanisms by playing a key role in creating the threshold for migraine attacks [89]. Some typical mechanisms in the action of magnesium include calcium channel blockade, neuroinflammatory blockade, N-methyl-D-aspartate (NMDA)-glutamate receptor blockade, effects on glutamate and nitric oxide synthesis, release and activity of serotonin receptors affinities, and endogenous hormone regulation [68]. Therefore, given that magnesium is present in all parts of the human body, it is involved in several process linked to migraine pathogenesis [68]. A large body of the literature confirmed a magnesium deficiency among migraine patients [90].

Peikert and colleagues [91] performed a randomized study in which, for 12 weeks, one group of patients was treated with magnesium and one group was the control. The group of patients that used magnesium had a greater reduction in the frequency of attacks in the last month of treatment compared to the placebo group (52.8% in the magnesium group and 34.4% in the control group). Unfortunately, adverse events were reported such as diarrhea and gastric irritation. Other studies confirmed these gastrointestinal problems as side effects in using magnesium. Moreover, magnesium pidolate is commonly used in the treatment of migraine in pregnant women, although some studies have shown adverse effects on fetal bone growth, thus recommending attention to the administration of magnesium [92,93,94,95].

The effectiveness of magnesium has been studied for both symptomatic treatment of migraine episode and prevention of migraine [96,97]. Some studies have also been conducted on tension-type headache in young patients, demonstrating magnesium effectiveness for the majority of patients [98,99]. Even if the preliminary results are very promising, more rigorous studies have to be designed to confirm the efficacy of magnesium for headache.

#### 3.2.5. Coenzyme Q10

Coenzyme Q10 (coQ10) is an essential cofactor of the electron transport chain and protects against mitochondrial collapse or degeneration by maintaining mitochondrial energy emission through proper electron transport along the respiratory chain [68,100]. Since patients affected by migraine showed a dysfunction of interictal mitochondrial in the occipital lobes, evidenced by spectroscopy of Magnetic Resonance Imaging (MRI), coQ10 could be beneficial in their treatment [101].

Two studies [102,103] showed promising results of the effectiveness of Coenzyme Q10, compared to the placebo, in the treatment of migraine, with minimal side effects: in one of these studies, some patients reported a cutaneous allergy which was not clinically worrying.

Positive results from several studies suggest that Coenzyme Q10 may be effective in migraine prophylaxis [101,102,103], even if higher quality studies are necessary to better assess the efficacy and safety of this treatment for migraine.

### 3.3. Behavioral Approaches

Behavioral approaches to headache consist of treatment interventions which are intended to modify maladaptive behaviors and thoughts that could increase headache-related burden and pain. There is evidence of the efficacy of behavioral interventions in the management of primary headache disorders in adults and children, particularly for migraine, both in episodic and chronic forms.

Behavioral approaches have proved to produce sizeable effects on headache frequency and to have a valuable impact on some patient-reported outcomes such as disability, quality of life, depression, anxiety, self-efficacy, and medication intake [4]. Recent reports show that the efficacy of behavioral approaches for headache makes it possible to consider them not just as alternative or complementary to pharmacotherapy, but as valid treatment options, with a comparable efficacy [104]. Moreover, the combination of pharmacological and behavioral therapies showed higher effectiveness compared to single approaches, i.e., compared to pharmacotherapy alone or behavioral therapies alone [104].

Different mechanisms of action of behavioral treatments, which reasonably act in combination, have been hypothesized. Behavioral treatments have an effect both on headache itself and on the concomitant conditions of distress and psychiatric symptoms that are frequent in chronic headache patients and commonly associated with poor prognosis. Behavioral treatments reduce stress, increase self-efficacy, and reduce the external locus of control. The combined effect of different factors could stimulate a change in the way patients perceive and experience pain, which in turn can lead to an improvement in symptoms [4,104,105]. At the biological level, these treatments seem to produce functional modifications in the brain areas and systems responsible for the perception and regulation of pain [4,106].

Recently, behavioral treatments are also becoming increasingly popular because conventional treatments are often considered by patients to be ineffective [4,19]. The use of behavioral treatments is common in clinical practice, mostly in specialized units such as headache centers that provide innovative treatments [4,19]. One of the barriers to the spread of these interventions is the need for neurologists or psychologists specialized in the use of behavioral techniques, which constitutes a cost, particularly if therapies are conducted individually and not in a group. Therefore, although there is much evidence of the effectiveness of behavioral approaches, these interventions are not widely used in clinical practice [105]. Moreover, the lack of a standardized treatment protocol in psychological treatment and the consequent diversity and heterogeneity of the specific protocols sometimes make it difficult to draw meaningful conclusions [104,107].

In the paragraphs below, different behavioral approaches will be presented. They include biofeedback, relaxation therapy, cognitive behavioral therapy (CBT), mindfulness-based therapy, and acceptance and commitment therapy (ACT).

#### 3.3.1. Biofeedback (BFB)

Biofeedback (BFB) is a therapeutic technique that allows an individual to learn to actively control and self-regulate their physiological responses that are usually beyond voluntary control [108]. Through the BFB method, the person is trained to connect their physiological and psychological processes, with the aim of improving their physical and mental health. BFB is frequently implied in the management of pain disorders, in which patients assume an active role in learning to manage their pain; this also turns into an improvement of self-regulation, coping cognitions, and changes of maladaptive psychophysiological responses [19,108,109].

Various research studies reported the efficacy of clinical BFB training and recommended it for treatment of primary headaches [108,110]. Based on the patient’s diagnosis, different types of BFB can be used [19] Some of the literature reviews reported recommendations for the use of BFB as an effective treatment for migraine and tension-type headache, producing a significant reduction in symptoms which also persisted at long-term follow-up [19,111].

Different studies involving both adults and children showed a reduction in headache frequency following BFB treatment [112,113], with a lower use of preventive medication [113]. BFB, added to traditional pharmacological therapy, resulted effective in the treatment of medication overuse headache, reducing the frequency of headache and medication intake [110].

Health funds usually do not cover or just partially cover the expenses of BFB treatment and this factor can hinder its diffusion in clinical practice [114].

#### 3.3.2. Relaxation Techniques

Relaxation techniques consist of tension and relaxation exercises of different muscle groups aimed at improving awareness of the sensations related to these processes; they are used to decrease sympathetic arousal and physiological responses to stress. Relaxation training includes several techniques and procedures [4,19]. The joint use of relaxation training with biofeedback or with cognitive behavioral therapy (CBT) can be employed to stimulate a relaxation response and teach relaxation skills [19,115].

Among relaxation techniques, the most popular and widely used for headache treatments are autogenic training (AT) and progressive relaxation training.

A systematic review on the effectiveness of autogenic training highlighted a significant reduction in headache by AT in the vast majority of the studies [116]. AT could be particularly suitable for TTH as it helps to control the overall level of activation, strongly associated with the emergence of this disorder [117]. Even a study using progressive relaxation training with children with TTH showed an improvement in headache frequency [118].

An advantage of the relaxation techniques is that they can be progressively learned by patients, becoming automatic and carried out without conscious effort. Therefore, patients can apply them in everyday situations, increasing the likelihood of long-term benefits. [19]. However, more research is needed on the effectiveness of relaxation therapies used alone, the most effective duration of training and practice, and the type(s) of headache for which they are most effective [116].

#### 3.3.3. Cognitive Behavioral Therapy (CBT)

CBT is a psychotherapeutic approach that includes both cognitive and behavioral strategies. CBT focuses first on recognizing and subsequently on modifying dysfunctional thoughts, behaviors, and responses to stress that may exacerbate, increase, or maintain headache [4].

Viewing headache in a biopsychosocial framework, as influenced by biological, cognitive, emotional, and environmental factors, supports the use of psychological treatment options, such as CBT [19,117,119].

The US Headache Consortium found evidence of the efficacy of CBT for migraine and TTH management [19,120,121,122]. Moreover, the combined used of CBT and pharmacological treatment may be more effective than both treatments alone [19,104].

Two recent reviews describing different categories of behavioral treatments reported the efficacy of CBT in improving headache outcomes [4,105]: different studies using CBT with adults and children found a reduction in the frequency of headache [4] as well as significant improvements in patient-reported outcomes, including disability, self-efficacy, quality of life, medication intake, depression, stress and anxiety, pain catastrophizing, pain acceptance, and pain coping [4,105]. CBT is found to have greater benefits in patients who have concomitant psychological or environmental problems that play a role in triggering headache, such as work-related stress, mood disorders, or adjustment problems [19]. Thus, it is also beneficial for the management of headache comorbidities of psychiatric illness [117].

There is much evidence that CBT effectively improves the cognitive, behavioral, and stress-related aspects of migraine [4,107,123]. However, the evidence regarding the effectiveness of CBT in reducing headache frequency is more variable [119]. A review by Harris [107] reported some studies that provide data in favor of the benefit of CBT for people with headaches and migraines, even with respect to a reduction in physical symptoms, but with mixed evidence.

From the available data, CBT appears to be an effective treatment option for headache. Additionally, since CBT promotes lifestyle changes and helps in the management of migraine symptoms, it can be effectively used to reduce the overall impact or burden of headache. However, further studies are needed, in particular to examine specific outcome measures [119].

#### 3.3.4. Mindfulness-Based Therapy

Mindfulness is a practice aimed at focusing on the present in a non-judgmental way, directing attention to body sensations, thoughts, and emotions, including pain. It enables people to concentrate on experiences from moment to moment. It also aims to promote an attitude of openness, curiosity, and acceptance, improving self-efficacy and decision making [4,122]. 

These interventions seem to effectively intervene on at least one headache outcome. In addition, various studies show that they are feasible and well tolerated, with a good degree of adherence to treatment by the participants. In recent years, mindfulness is becoming increasingly popular, since it is proving effective in treating headaches and pain in general [105].

Regarding mindfulness, different studies reported a decrease in the frequency of headache in patients affected by CM and CTTH [124,125]. Those studies that did not find a reduction in headache frequency found a reduction in headache duration [126].

In general, some recent reviews and meta-analysis evidenced the efficacy of mindfulness for the management of migraine and other severe headaches [121,127], suggesting that it may produce effects comparable to pharmacological treatment [98]. Moreover, mindfulness proved to impact disability, medication consumption, self-efficacy, quality of life, depression, and anxiety [4]. There is also evidence of the long-term durability of the effects of mindfulness-based therapy [128].

Unlike other well-established behavioral therapies for headache, mindfulness is emerging as a potentially effective treatment [129], but the evidence is sometimes still contrasting as some reviews outlined no improvement of headache condition after mindfulness-based treatment [130]. Future studies will increase the understanding of the impact and long-term effects of mindfulness on specific headache disorders [121,122,131].

#### 3.3.5. Acceptance and Commitment Therapy (ACT)

Acceptance and commitment therapy (ACT) is a psychological and psychotherapeutic intervention that uses acceptance and mindfulness strategies together with strategies of engagement in action and behavior modification [132]. This allows for an increase in psychological flexibility, which means being fully in contact with the present moment and acting effectively in the presence of difficult or interfering private events, such as pain [133,134]. While mindfulness consists of developing awareness of bodily sensations and of the present with a non-judgmental attitude, ACT focuses on experiencing pain, without trying to control it. ACT aims to increase valued action, which means to encourage people to persist in activities considered important even in the presence of stressful or critical conditions such as pain [105,135]. 

As regards the use of ACT in the management of headache, two systematic reviews have found improvements in patient-reported outcomes and in the affective perceptions of pain and anxiety [133,136]. As well as mindfulness, ACT is receiving increasing interest in the study of headache management because it showed evidence of reduction in chronic pain and it is a feasible and well tolerated treatment [105,134].

## 4. Discussion

With this review, we summarized the available recent literature on the use of non-pharmacological approaches as aids for the treatment of headache disorders, namely non-invasive neuromodulation, nutraceutical, and behavioral approaches [137]. Taken as a whole, the results show that these approaches are usually efficient in reducing headache frequency and prevent the overuse of symptomatic medications; therefore, after a deep clinical assessment, they could be used particularly in some kind of patients, such as pregnant women, children and adolescents, and in all those patients that, for different reasons, cannot be prescribed specific medications due to comorbidity profile or risk of overuse.

As far as neuromodulation is concerned, various scientific evidence has highlighted numerous advantages: ease of application, infrequency of adverse effects, and high tolerability. However, the economic costs related to the technological equipment implied for neurostimulation hinder the wide implementation of these treatments in the care system. In addition, the lack of clearly understanding the mechanism of action and the necessity of specialized neurologists who know how to use these techniques make their clinical application difficult [134]. Hopefully, future trials will confirm the first encouraging results and will allow these techniques to be used on an increasingly large number of patients [138].

Concerning nutraceuticals, even if the number of adequately powered studies is still small and there is difficulty in translating the results into daily clinical application, several studies show promise as potential treatments for headache.

Furthermore, patient comorbidities should be taken into account because some of these therapies have been effectively used in the treatment of other conditions, thus they can provide a dual benefit. For instance, using nutraceuticals in women affected by menstrual migraine and premenstrual syndrome can help decrease pain related to these conditions [17].

Due to limited data, it is important to be careful in the choice of these interventions in clinical practice, but a physician trained in the use of nutraceuticals could provide better care to their patients [17].

In regard to behavioral approaches, one of the main advantages is that they can be used in combination with pharmacological treatments. Behavioral approaches have an important role in reducing headache frequency and in producing improvements in other patient-reported outcomes that significantly contribute to the prognosis, such as disability and intake of acute medications [4]. Furthermore, active patient involvement would appear to be a self-help technique that could potentially lead to long-term effects [80]. Unfortunately, the lack of standardized treatment protocols leads to very heterogeneous results. More studies are needed to ameliorate the knowledge about the behavioral approaches’ mechanisms of action and to guide clinicians in choosing these approaches in their daily practice [104].

The main strength of this review of the literature is the fact that it analyzes the three main non-pharmacological interventions for the treatment of headache and their feedback in clinical practice. This has allowed us to have a more complete view on all the approaches that can be used as a support or as an alternative to pharmacological treatment, especially for the categories of patients considered more fragile, such as pregnant women and children.

As for limitations, it certainly must be taken into account that our review is not a systematic review and therefore, some studies may not have been considered.

Furthermore, research often focuses on direct clinical trial results but not on long-term results, which could also provide more reliable and generalizable data for building new protocols.

## 5. Conclusions

In recent years, much of the literature has focused on the effectiveness of non-pharmacological approaches for the treatment of headaches, which deserve particular attention both in research and in clinical practice.

These approaches, which include non-invasive neuromodulation, nutraceuticals, and behavioral approaches, are increasingly seen as valid treatment options, particularly for specific conditions, such as the presence of medication overuse or contraindications to drug treatment, and for specific patient categories, such as pregnant women and pediatric populations.

However, more investigation is needed to also investigate the long-term effects, in order to better understand the effectiveness of these approaches, even for different types of headache. In this way, it will be possible to obtain more standardized and generalizable protocols to larger patient populations.

## Data Availability

Not applicable.
